# Rotary Instrument–Induced Soft Palatal Laceration During Dental Restorative Treatment: A Case Report

**DOI:** 10.7759/cureus.104125

**Published:** 2026-02-23

**Authors:** José Emiliano González Flores, Araceli Pérez González, Raymundo Canseco Lopez, Alfonso Sandoval Polito

**Affiliations:** 1 Department of Surgery, Tecnológico de Monterrey, Campus Ciudad de Mexico, Mexico City, MEX; 2 Department of Plastic and Reconstructive Surgery, Dr. Manuel Gea González General Hospital, Mexico City, MEX; 3 Department of Dentistry, Universidad Nacional Autónoma de México, Mexico City, MEX

**Keywords:** dental restorative procedures, dental trauma, high-speed dental handpiece, iatrogenic injury, oral soft tissue injury, palatal laceration, posterior oral cavity, rotary instruments, secondary intention healing, soft palate

## Abstract

Iatrogenic soft tissue injuries are recognized complications of dental procedures, most commonly affecting the tongue, buccal mucosa, and gingiva. However, trauma involving posterior palatal structures remains rarely documented. This case report describes a deep soft palatal laceration sustained during posterior restorative dental treatment following accidental contact with a high-speed rotary instrument. The injury resulted in mucosal and muscular disruption requiring surgical repair, which was subsequently complicated by early suture dehiscence due to the high-mobility nature of the soft palate. Healing progressed by secondary intention, ultimately achieving complete mucosal re-epithelialization with residual fibrotic scarring but no functional impairment. This case highlights the vulnerability of posterior palatal tissues within confined operative fields and underscores the importance of meticulous soft tissue protection, operative control, and postoperative monitoring. Increased recognition of such complications may inform preventive strategies and guide future research on rotary instrument-related soft tissue injuries in restorative dentistry.

## Introduction

Iatrogenic trauma to oral soft tissues represents a recognized complication of dental procedures, encompassing a spectrum that ranges from minor mucosal abrasions to deep lacerations requiring surgical intervention. Such injuries may arise from mechanical, thermal, or chemical factors associated with routine dental care. The inadvertent or improper use of dental instruments, including rotary handpieces, electrosurgical units, and other operative devices, has been well documented as a potential source of mucosal damage, capable of producing ulceration, epithelial sloughing, and localized tissue necrosis [1].

Restorative dental treatment routinely necessitates the use of high-speed rotary instrumentation for caries removal and cavity preparation. While indispensable for contemporary operative dentistry, these devices function within confined intraoral operative fields where visualization and soft tissue retraction may be inherently limited, particularly in posterior anatomical regions. Under such conditions, adjacent mucosal structures remain vulnerable to inadvertent trauma during operative manipulation, especially when patient movement, restricted access, or inadequate tissue protection are present [2].

Although the tongue, buccal mucosa, and gingival tissues constitute the most frequently affected sites in iatrogenic dental injuries, involvement of posterior palatal and oropharyngeal structures has been reported only rarely in the literature. Trauma affecting the soft palatal pillar carries particular clinical relevance given its proximity to the oropharynx, potential for hemorrhage, risk of secondary infection, and implications for swallowing discomfort and airway safety. The present report describes a case of rotary instrument-induced soft palatal laceration sustained during posterior restorative dental treatment, highlighting a scarcely documented anatomical site of injury and underscoring the need for heightened operator awareness and meticulous soft tissue protection during confined posterior operative interventions.

## Case presentation

A 25-year-old male patient with a past medical history significant for unilateral left cleft lip and palate repair presented following an iatrogenic oropharyngeal soft tissue injury sustained during routine dental treatment. The patient was otherwise systemically healthy, with no history of coagulopathy, immunosuppression, or chronic medication use.

**Figure 1:**
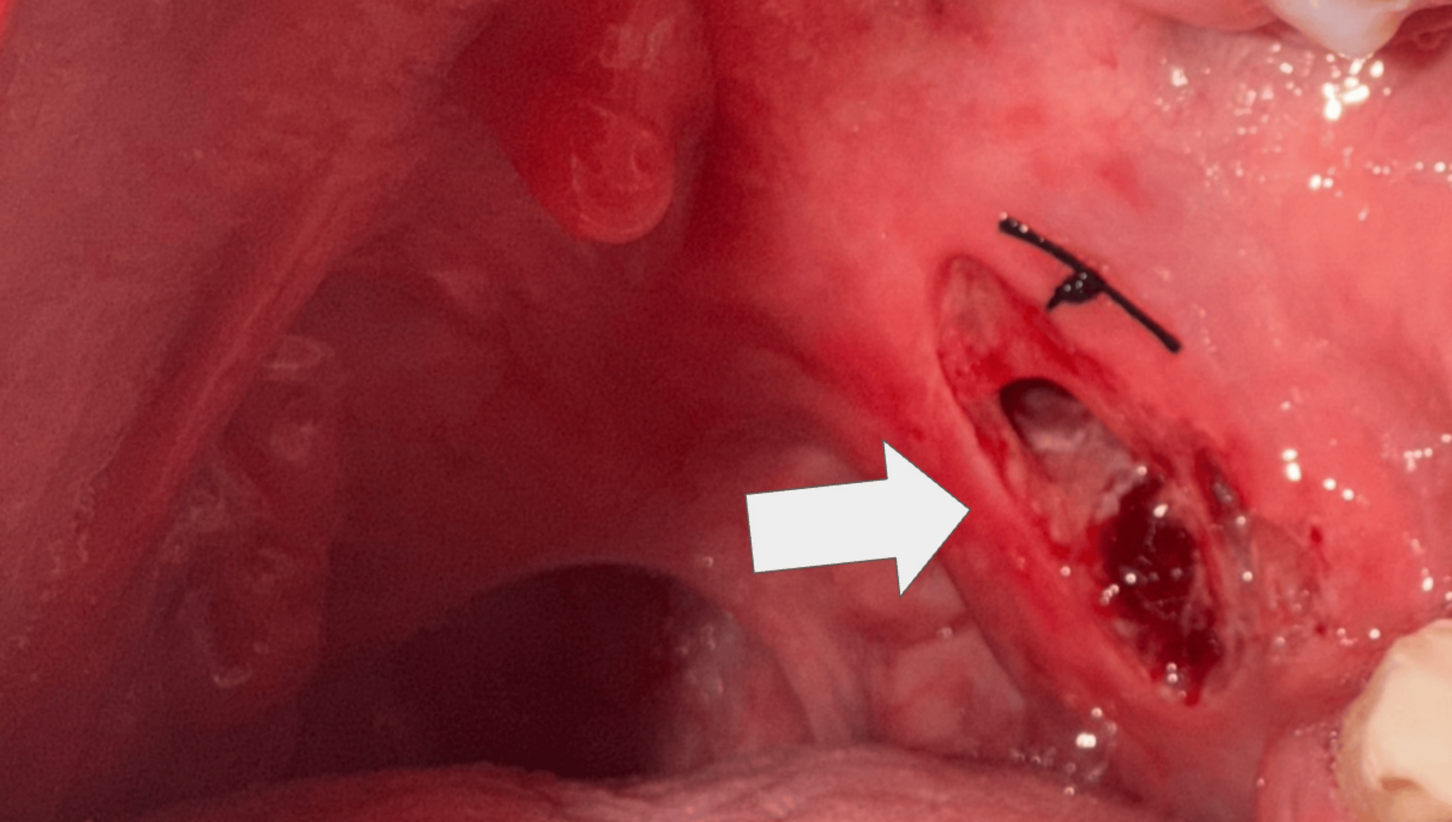
Acute intraoral view of the iatrogenic soft palatal injury Clinical photograph demonstrating a deep linear laceration involving the posterior soft palate and tonsillar pillar region following accidental contact with a high-speed rotary dental instrument during restorative treatment. Partial mucosal loss with exposure of underlying muscular tissue is observed, accompanied by active bleeding. An initial superficial suture placed at the primary care setting can be seen superior to the wound margins without adequate tissue approximation.

The patient presented for dental consultation for restorative management of dental caries involving the maxillary third molar. During caries removal using a high-speed rotary instrument, inadvertent contact between the rotating bur and the posterior palatal mucosa occurred, resulting in a deep soft tissue laceration. The procedure was discontinued, and an initial attempt at wound closure was performed at the primary care dental setting before referral. Initial suturing was undertaken; however, definitive tissue approximation was not achieved (Figure 1). Detailed operative records, including isolation techniques and intraoperative procedural specifics, were not available at the time of specialist evaluation, as the injury occurred at an external facility. The patient was subsequently referred for further evaluation and management of the soft tissue injury.

The patient was referred for specialist evaluation approximately five hours after the injury due to persistent deep bleeding and severe pain, rated 10 out of 10 on the visual analog scale. Plastic surgery consultation was obtained on an urgent basis. Clinical assessment included a detailed intraoral examination to determine wound depth, mucosal loss, muscular involvement, and airway safety. Based on the extent of tissue disruption and active bleeding, definitive layered wound repair was recommended under local anesthesia. Intraoral examination revealed an extensive linear laceration involving the posterior soft palate and tonsillar pillar region. The wound measured approximately 7 cm in length and 2 cm in width, with loss of mucosal integrity and involvement of the underlying muscular layer. Active bleeding was noted, with surrounding edema and erythema. No airway compromise was identified at the time of examination.

Given the clear traumatic mechanism and characteristic intraoral findings consistent with acute soft tissue injury, no biopsy, imaging, or histopathological testing was indicated. Diagnosis of iatrogenic rotary instrument-induced soft palatal laceration was established clinically. No imaging studies were required.

**Figure 2:**
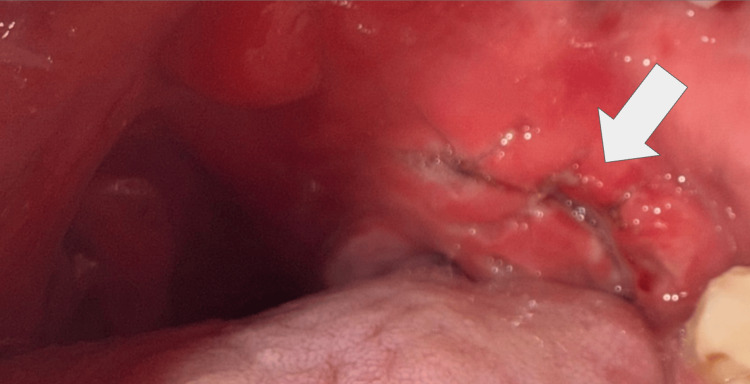
Post-repair intraoral view following secondary wound evolution Clinical photograph demonstrating the posterior soft palatal region after initial surgical repair. Evidence of partial wound dehiscence is observed, with loss of suture fixation in a high-mobility anatomical zone. Early granulation tissue formation and fibrin deposition are present along the wound bed, without signs of active hemorrhage or infection. Findings are consistent with progression toward secondary intention healing.

Definitive wound management was performed by a plastic surgeon under local anesthesia. Copious irrigation was performed, followed by layered wound approximation using interrupted non-resorbable black silk sutures to achieve mucosal edge stabilization and hemostasis (Figure 2). Hemostasis was achieved, and postoperative care instructions were provided.

Analgesic management included non-steroidal anti-inflammatory drugs, with the patient reporting the need for sublingual ketorolac every eight hours for pain control. A follow-up dental evaluation was later conducted to monitor healing progression.

The decision to pursue primary closure was based on intraoperative evaluation of tissue viability, vascular perfusion, and wound depth. Despite the patient’s history of cleft lip and palate repair, the surrounding palatal tissues demonstrated adequate structural integrity to support layered approximation. Primary closure was selected to achieve hemostasis, protect the exposed muscular layer, and promote functional healing. Alternative management through secondary intention healing was considered; however, immediate tissue stabilization was deemed preferable in the acute traumatic setting.

Due to continuous soft palate mobility, suture dehiscence occurred within 48-72 hours post-repair. The sutures remained in place for approximately three days before complete loss of fixation. The patient experienced severe pain, rated 10/10, during the acute postoperative phase, accompanied by persistent bleeding for up to three days following injury.

**Figure 3:**
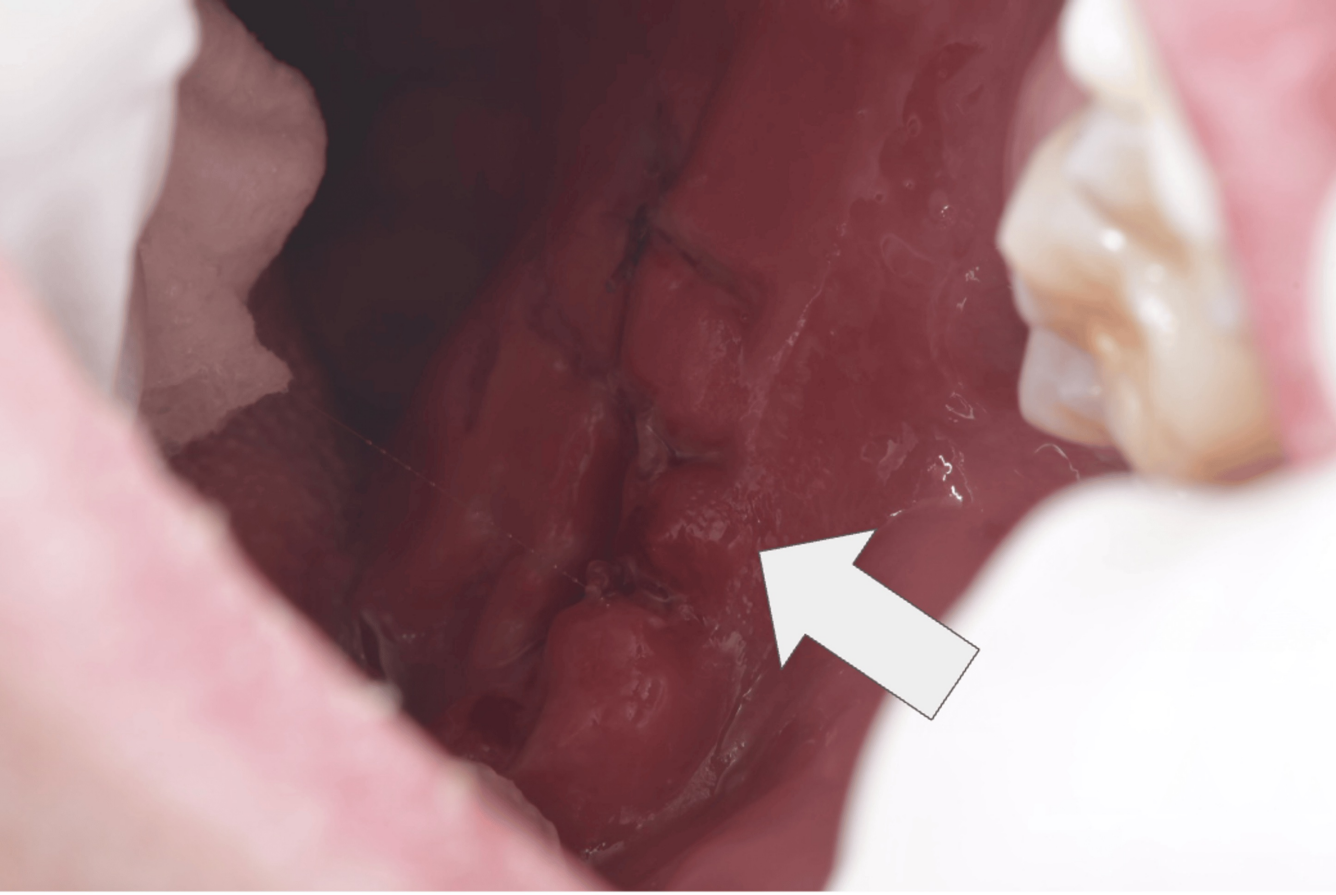
Late postoperative intraoral view demonstrating fibrotic scar formation Clinical photograph obtained during follow-up evaluation showing complete mucosal re-epithelialization of the posterior soft palatal region. Dense localized fibrotic scar tissue is observed at the prior laceration site, consistent with healing by secondary intention in a high-mobility anatomical area. No evidence of dehiscence, infection, or residual mucosal defect is noted.

Subsequent evaluation by a dental specialist recommended secondary intention healing. Progressive granulation tissue formation and mucosal re-epithelialization were observed over time in Figure 3, without evidence of infection, airway compromise, or long-term functional deficit at follow-up. At the time of the most recent follow-up, the patient remained free of functional impairment. No dysphagia, speech alterations, airway compromise, or limitations in oral intake were reported. Intraoral examination demonstrated complete mucosal re-epithelialization with adequate wound closure. However, a localized area of significant fibrotic scar tissue was noted at the site of injury, consistent with secondary intention healing in a high-mobility anatomical region.

## Discussion

Iatrogenic trauma to oral soft tissues constitutes a recognized complication of dental practice, encompassing a broad spectrum of lesions ranging from superficial mucosal abrasions to deep lacerations involving underlying muscular structures. Such injuries may result from mechanical, thermal, or chemical mechanisms associated with routine dental interventions. Improper or inadvertent use of dental instruments, including rotary handpieces and surgical burs, has been consistently identified as a primary etiologic factor in mucosal injury, particularly when operative field control is compromised [1].

Rotary dental instrumentation plays an indispensable role in restorative dentistry; however, its high rotational velocity confers significant potential for soft tissue damage upon direct contact. Experimental and clinical investigations have demonstrated that rotary burs are capable of producing measurable lacerations in soft tissues, including deep structural disruption when protective barriers are inadequate [2]. The capacity of these instruments to inflict direct tissue injury underscores the importance of meticulous technique and soft tissue retraction during operative procedures.

Anatomical location represents a critical determinant of injury risk. While the majority of reported iatrogenic soft tissue lesions involve the tongue, buccal mucosa, and gingival tissues, posterior oral cavity structures remain less frequently described sites of trauma [2,3]. The proximity of soft palatal tissues to posterior maxillary operative fields, particularly during third molar or restorative interventions, creates a confined working space in which visualization and instrument control may be inherently limited. Studies evaluating lingual nerve injury during third molar surgery further highlight the vulnerability of posterior soft tissue structures to inadvertent operative trauma, reinforcing the concept of this region as a high-risk anatomical zone [4].

Management of extensive mucosal lacerations depends on wound size, depth, and anatomical mobility. Primary closure is generally recommended for large or deep soft tissue injuries to promote hemostasis and optimize healing. High-mobility regions such as the soft palate, however, present unique surgical challenges. In patients with prior cleft palate repair, scar tissue characteristics, altered vascularity, and tissue elasticity must be considered when selecting a closure strategy. In the present case, intraoperative evaluation demonstrated adequate perfusion and local tissue mobility to support layered primary approximation in the acute setting. Alternative approaches, including healing by secondary intention or the use of soft tissue grafts and biologic adjuncts, may be considered in cases of compromised vascularity or extensive tissue loss. Given the traumatic mechanism, exposed muscular layer, and active bleeding, primary closure was initially selected to achieve hemostasis and structural stabilization. Continuous movement associated with swallowing, phonation, and respiration may compromise suture stability and predispose to early wound dehiscence, as observed in the present case. When primary closure fails, healing by secondary intention remains a viable alternative, often resulting in satisfactory mucosal regeneration despite prolonged recovery periods [2].

The clinical course observed in this patient further demonstrated that secondary intention healing within the soft palate can culminate in complete mucosal re-epithelialization, albeit with residual fibrotic scar formation. Fibrosis in such contexts represents a predictable reparative response following deep tissue injury, particularly in areas subjected to constant mechanical stress. Importantly, despite the severity of the initial laceration, no long-term functional impairment was identified, including swallowing dysfunction, speech alteration, or airway compromise.

The posterior mandibular and oropharyngeal operative field represents a well-documented risk zone for iatrogenic injury due to limited visibility and close anatomical relationships. From a preventive standpoint, meticulous operative field control, adequate visualization, and careful soft tissue retraction are critical when performing restorative procedures in anatomically constrained posterior regions. The use of protective barriers, controlled rotary instrumentation, and patient stabilization measures may reduce the risk of inadvertent mucosal injury. Heightened awareness of posterior palatal vulnerability is essential, particularly in confined operative fields where limited access and restricted visibility may increase soft tissue exposure to rotary devices. Previous investigations on lingual nerve trauma during third molar surgery have emphasized the vulnerability of adjacent soft tissues to rotary instrumentation and surgical manipulation [5,6].

This case contributes to the limited body of literature describing deep posterior palatal lacerations of iatrogenic dental origin. Its educational value lies in the clear mechanistic association between rotary instrumentation and soft palatal trauma, the documentation of wound evolution through sequential imaging, and the demonstration of both surgical and conservative healing pathways. Increased awareness of this potential complication may enhance preventive operative strategies, particularly in anatomically constrained posterior restorative settings. The principal teaching points highlighting the mechanism of injury, anatomical risk factors, and management considerations are outlined in Table 1.

**Table 1:** Key teaching points derived from the present case and supporting literature

No.	Teaching point	Clinical relevance	Reference
1	Iatrogenic soft tissue injuries are recognized complications of routine dental procedures.	Even standard restorative interventions carry risk when operative control is limited.	[1]
2	High-speed rotary instruments can produce deep mucosal and muscular lacerations upon direct contact.	Demonstrates the mechanical injury potential of dental burs.	[3]
3	Posterior oral cavity structures represent high-risk zones due to limited visibility and restricted operative access.	Highlights anatomical vulnerability during third molar and posterior restorative procedures.	[4]
4	Primary closure in high-mobility regions such as the soft palate carries increased risk of wound dehiscence.	Important for surgical planning and postoperative expectations.	[2]
5	Secondary intention healing may achieve satisfactory functional outcomes despite residual fibrosis.	Supports conservative management when re-suturing fails.	[2]

## Conclusions

Iatrogenic soft palatal injury represents a rare but clinically significant complication of restorative dental procedures involving high-speed rotary instrumentation. This case underscores the capacity of rotary dental burs to produce deep mucosal and muscular lacerations within anatomically confined posterior operative fields, particularly when soft tissue protection, retraction, and visualization are limited. Despite the severity of the initial injury and the occurrence of early wound dehiscence, secondary intention healing may result in satisfactory functional recovery, although residual fibrotic changes may persist.

From a clinical and preventive standpoint, this report reinforces the importance of strict adherence to operative safety principles during posterior dental interventions. Meticulous operative field control, appropriate soft tissue isolation and retraction, controlled instrument handling, and heightened awareness of regional anatomical vulnerability are essential measures to minimize the risk of iatrogenic soft tissue trauma. Early recognition of deep oropharyngeal injury and timely specialist referral remain critical to optimizing patient outcomes.

Future investigations should aim to further characterize the incidence, anatomical risk factors, and optimal management strategies of iatrogenic posterior oral soft tissue injuries. Multicenter observational studies and procedural safety analyses may provide valuable insight into preventive protocols and evidence-based management pathways for rotary instrument-related complications.
